# Behçet syndrome as a construct

**DOI:** 10.3906/sag-2002-145

**Published:** 2020-11-03

**Authors:** Hasan YAZICI*

**Affiliations:** 1 Department of Behçet Disease Resarch Center, Cerrahpaşa And Academic Hospital, İstanbul University, İstanbul Turkey

## Abstract

We still do not know the cause(s) of Behçet syndrome. Most probably several, separate disease mechanisms are involved. I, like some others, propose we call it not a disease but a syndrome, a construct with a list of strong and weak elements. I like to think that this frank admission of our ignorance of its cause(s) will be an important semantic stimulus for more meaningful research.

The pathologic entity first described by Hulusi Behçet in 1937 [1] is more commonly called a disease rather than a syndrome, as some students of this condition, like me, like to call it. Webster’s dictionary [2] defines a disease as a disorder of structure or function in a human, animal, or plant, especially one that produces specific signs or symptoms or that affects a specific location and is not simply a direct result of physical injury while this authoritative source describes a syndrome as a group of signs and symptoms that occur together and characterize a particular abnormality or condition. What is lacking in these definitions is the place of causality. While a disease may or may not have a cause, the medical profession most commonly associates a syndrome with an unknown causality. Finally a construct is a theoretical entity [2] and a hypothesis that our mind makes. My last comment in this introduction in the light of these quotes from Webster’s, I am sure boring to some, my title would come as a tautology. Well, it is intended to be. I simply want to emphasize that we still do not what causes Behçet syndrome (Bsy) and need to be a splitter to decipher the cause (s) of the construct we have made thus far. This is not only an intellectual exercise but to better to take care of our patients.

As we have recently summarized the construct of Bsy has strong and weak elements [3] like any other construct before it becomes a construction. I propose that there are 4 strong and 4 weak such elements.

The strong elements: Frequent oral ulceration is a strong element the absence of which very strongly suggests another condition. On the other hand, the remaining 3 elements are very specific for Bsy. Our group had initially shown this many years ago, where in factor analysis it had shown this as a disease defining aspect [4]. More recently it was formally shown that some peculiar characteristics of retinal disease enable ophthalmologists to classify even from retinal photographs of Bsy patients without knowing what else a patient has [5]. These characteristics include a smooth-layered hypopyon, superficial retinal infiltrate with retinal hemorrhages, and branch retinal vein occlusion with vitreous haze.

The genital ulcerations were the most specific feature in the classification criteria we had formulated some years ago, still widely used [6]. Interestingly, in the male, they are almost always located on the scrotum, the skin of which is probably the most testosterone sensitive tissue in the body. In Behçet syndrome endemic areas noting genital scars, unless the patient gives another specific reason for their presence is a very specific finding.

Major vascular disease is another strong element. Bsy is almost unique among the vasculitides to involve vessels of all sizes. If one considers only deep and superficial vein thromboses (DVTs and SVTs) about 1/3 of the patients have them. The involvement of larger vessels like the vena cavae and pulmonary arteries is around 5%. On the other hand, the vasculitis in Bsy is peculiar in that may be different sites of the vascular tree are involved at the same time and this is particularly true for venous disease. For example the presence of dural sinus thrombi, pulmonary artery aneurysms (reminding ourselves that pulmonary arteries are very similar to veins both structurally and by their content), venae caval disease, DVT and SVT frequently cluster in the same patient [7]. This is a pattern not perhaps at all observed in other vasculitides.

The pattern of nervous system involvement is the last strong element. To start with peripheral neuropathy commonly seen in other primary and secondary vasculitides is most uncommon. The parenchymal, making up of 80% of central nervous system disease, the remainder is dural sinus thrombi, is a unique vasculopathy of patches of inflammatory cell accumulation characteristically in the brain stem. We call this a vasculopathy since a frank vascular wall inflammation, again common among other vasculitides, is not seen.

The weak elements: These are made up of the distinctly peculiar geographic distribution, the different clusters of disease subsets [8,9], the singular difficulty to tell intestinal involvement in Bsy from Crohn`s disease unless our patient has other unique extra-intestinal manifestations and finally the differing efficacy of different drugs in different organ manifestations (for example, colchicine has almost no effect on eye disease while it is rather effective in arthritis and e. nodosum). These weak elements suggest that we are dealing with a syndrome rather than a disease in what was described by Hulusi Behçet back in 1937 [1] and most probably more than one disease mechanism is at hand.

Future work: There is good consensus among the disease, syndrome or construct callers that we still do not know the cause(s) of Bsy. Here, I humbly suggest that we give much more attention in our research efforts to what earlier I mentioned as the strong elements. In this line, I am gratified to see that recently 2 separate, one from ours, groups reported that venous wall is diseased among Bsy patients even when they have no clinical vascular disease [10,11]. However this is obviously quite preliminary. We need to know much more about the pathology in the venous wall in greater depth, in its biochemical, inflammatory/immunologic and genetic aspects soberly reminding ourselves that the venous wall has always been the stepchild of the basic researchers as compared to the arterial wall.

Finally, we should in general aim at deciphering how Bsy syndrome differs from other nosologic entities rather than how it goes cozy with them [12,13]. This is why in this short communication I initially gave a somewhat boring emphasis on semantics. I simply wanted to emphasize that our admitted ignorance should be our semantic stimulus to understand Bsy better.
